# An analysis on history of childhood adversity, anxiety, and chronic pain in adulthood and the influence of inflammatory biomarker C-reactive protein

**DOI:** 10.1038/s41598-023-44874-1

**Published:** 2023-10-21

**Authors:** Danielle E. Dalechek, Line Caes, Gwenne McIntosh, Anna C. Whittaker

**Affiliations:** 1 Faculty of Health Sciences and Sport, University of Stirling, Stirling, FK9 4LA UK; 2 Division of Psychology, Faculty of Natural Sciences, University of Stirling, Stirling, FK9 4LA UK

**Keywords:** Stress and resilience, Biomarkers, Risk factors

## Abstract

Despite a link between adverse childhood experiences (ACEs) and anxiety, the role of anxiety in the pathway to chronic pain is unclear. Potentially, inflammatory biomarkers such as C-reactive protein (CRP) are involved. Objectives were to (1) examine relationships between reported ACEs, anxiety, and chronic pain, and (2) assess associations between ACEs, anxiety, and CRP levels and between CRP and chronic pain. Data from 24,172 adults who participated in the UK Biobank were used to conduct Poisson regressions to assess relationships between ACEs, anxiety, and chronic pain. For participants with CRP data who met the inclusion criteria (n = 2007), similar models were run between ACEs, anxiety, and CRP, and CRP and chronic pain. For objective 1, three statistically significant interactions were found to predict pain: frequency of physical abuse x reported muscular symptoms during anxiety (*p* = 0.01); frequency in which they felt hated x having discussed anxiety with a professional (*p* = 0.03), and reported frequency of sexual abuse x difficulties relaxing during anxiety attacks (*p* = 0.03). For objective 2, frequency of sexual abuse and informing a professional about anxiety significantly interacted to predict elevated CRP. For correlations, the largest was between CRP and the number of times pain was reported over the years (*p* = 0.01). Finally, ACEs (physical abuse, sexual abuse, and whether taken to a doctor) significantly interacted with CRP to predict pain. This study suggests mechanisms of the impact of ACEs on chronic pain may include inflammation and anxiety, which warrants further study.

## Introduction

During traumatic and tense experiences, the brain is in a heightened state of stress, which can have negative impacts over time^1^. Existing research has focused on behavioral responses, emotional development, and mental and physical health after stress, but research addressing a direct link between anxiety as a response to trauma and the experience of chronic pain in adults is limited. The long‐term impacts of the hyperarousal experienced in situations of high anxiety and stress are not fully understood when considering pain, although studies on depression and posttraumatic stress disorder (PTSD) have examined how these conditions are associated with pain experiences^2^. For example, one study^2^ found that specific pain coping strategies and depressive symptoms had a partially mediating effect on the relationship between PTSD and both pain interference and severity. Prior studies of depression have also indicated a higher prevalence of inflammation, particularly in adults who experienced early life adversity^3^.

Additionally, a large body of research has examined whether early life adversity or childhood adverse experiences (ACEs) contribute to the development of chronic pain in adulthood. The concept that childhood maltreatment predisposes individuals to develop pain conditions has been especially prevalent for disorders that involve unexplained pain paired with psychological complaints such as anxiety. One mechanism identified as a potential mediator of the relationship between ACEs and chronic pain in adulthood is dysregulation of the hypothalamic-pituitary-adrenocortical (HPA) axis^4^. The HPA axis is a complex system of neuroendocrine pathways involving the hypothalamus, anterior pituitary gland, and adrenal gland that function to maintain physiological balance via metabolism, immune responses, and the autonomic nervous system^5^. Experiencing ongoing ACEs may influence both the degree and the direction of HPA axis abnormalities^6^. These abnormalities can then affect other biological processes such as inflammation^7,8^.

Specific inflammatory processes, as reflected by blood-based markers such as C-reactive protein (CRP)—a protein that responds to inflammatory stimuli by triggering cellular reactions—could also be involved in the biological component effects of ACEs. Elevated levels of proinflammatory cytokines like interleukins—a downstream product of CRP signaling, and acute-phase proteins including CRP—have been observed in the plasma of individuals who have experienced ACEs^9^. Meta-analyses of cross-sectional studies have also confirmed the association of higher inflammation with traumatic experiences^9^.

Further, longitudinal studies have provided evidence supporting a bidirectional association: elevated inflammation may contribute to trauma symptoms, which in turn contribute to more elevated inflammation. Trauma symptoms are also known to be associated with several chronic diseases that have a confirmed inflammatory component, ranging from cardiovascular to chronic pain^9^. Neuropeptides, which can exhibit a variety of inflammatory effects, modulate neural activity and other tissues, including the gut and heart^10^, and it is possible that ACE exposure could impact normal neuropeptide synthesis. This can lead to disruptions in HPA axis development, which may then lead to abnormal physiological functioning in adulthood and increase the risk for disease and chronic problems in adulthood^5^.

Beyond their link with trauma symptomology, proinflammatory cytokines at higher levels may also influence neurotransmission. This can result in an altered production of neurotransmitters, which may be associated with specific psychiatric symptoms, and these cytokines could also impact neurocircuitry, causing changes in an individual’s levels and functionality of motivation, alarm-based responses, and anxiety^9^. However, studies of inflammation and anxiety symptoms remain scarce^11^. Indeed, most prior studies on anxiety were typically limited to the context of psychology and exclude a biological component^12^. In the last decade, neuroimaging studies have improved insights of brain pathways potentially mediating an anxiety–chronic pain interaction, with multiple brain areas implicated including the amygdala, anterior cingulate cortex, and insular cortex^12^.

There remains a lack of understanding of the complex pathways linking ACEs to poor adult health outcomes and exactly how those pathways may vary across different adverse adult outcomes, such as chronic pain versus heart disease^13^. In particular, despite a clear link between anxiety and ACEs, as well as between anxiety and chronic pain, the role of anxiety in the pathway between ACEs and chronic pain is not yet understood. Limited evidence suggests that ACEs can influence biophysiological functioning in adults with chronic pain. However, whether the link between ACEs and biophysiological functioning is unique to individuals with chronic pain or can be accounted for or exacerbated by current psychopathologies or inflammatory biomarkers remains unclear. Understanding the potential mechanisms underlying the ACE-inflammation association could potentially lead to important implications for systemic-level dysfunction after early life adversity and resulting health outcomes in adulthood. The purpose of the current analysis was to examine the relationships between reported ACE(s), anxiety, and levels of CRP in adults with chronic pain.

### Primary objectives

The first objective of this study was to investigate the relationship between ACEs, anxiety diagnosis, and chronic pain experience. A second objective was to explore whether ACEs, anxiety, and chronic pain experiences in adults are also associated with the inflammatory biomarker CRP and examine its role in the ACE-anxiety-pain pathway. It was hypothesized that there would be a significant association between ACEs, anxiety, and chronic pain experiences, and that CRP may also relate to some or all of these variables.

## Methods

### Data source

The dataset used—the UK Biobank (UKB)—holds an unprecedented amount of data on half a million participants aged 40–69 years (with a roughly even number of men and women) recruited between 2006 and 2010 throughout the UK. This retrospective analysis involved deidentified case data from 25,249 adults who participated in the UKB and met the inclusion criteria for this study. General inclusion for the current analyses required adults with at least one chronic pain measure (from Table 1), reported history of ACEs, and reported anxiety. Participants were excluded if they reported no anxiety, no chronic pain, and no ACEs.

**Table 1:** Factors of Interest from the UKB.

Biobank ID	Description	Category*	Response type
**1018**	**Mental health (via online follow-up questionnaire)**		
20487	Felt hated by family member as a child	ACE/Traumatic events	Scale (never–very often)
20488	Physically abused by family as a child	ACE/Traumatic events	Scale (never–very often)
20489	Felt loved as a child	ACE/Traumatic events	Scale (never–very often)
20490	Sexually abused* as a child	ACE/Traumatic events	Scale (never–very often)
20491	Someone to take to doctor when needed as a child	ACE/Traumatic events	Scale (never–very often)
20428	Professional informed about anxiety	Anxiety	Yes/no
20417	Tense, sore, or aching muscles during worst period of anxiety	Anxiety	Yes/no
20515	Recent trouble relaxing	Anxiety	Scale (not at all–nearly every day)
**1003**	**Self-reported medical conditions (via touchscreen)**		
3571	Back pain for 3 + months	Chronic pain	Yes/no
4067	Facial pains for 3 + months	Chronic pain	Yes/no
2956	General pain for 3 + months	Chronic pain	Yes/no
3799	Headaches for 3 + months	Chronic pain	Yes/no
3414	Hip pain for 3 + months	Chronic pain	Yes/no
3773	Knee pain for 3 + months	Chronic pain	Yes/no
3404	Neck/shoulder pain for 3 + months	Chronic pain	Yes/no
3741	Stomach/abdominal pain for 3 + months	Chronic pain	Yes/no
**717**	**Biomarkers**		
30710	C-reactive protein	Blood biochemistry	Instances 0–1 (mg/L)

UKB, UK Biobank. *The category of childhood “traumatic events” as defined by the UKB was our marker of ACEs or early life adversity. Sexual abuse was written as molestation in the questionnaire, but we have updated abuse throughout this manuscript for consistency.

Each objective had a different sample size due to exclusion criteria and availability of records. The way each exclusion criterion was interpreted was different for each. If the participant was excluded due to no anxiety, it meant they did not meet one of the three included anxiety measures (had informed a professional about their anxiety, reported muscular symptoms related to anxiety episodes, or reported trouble relaxing in relation to anxiety episodes). For exclusion due to no chronic pain, the person never reported pain of any type during their visits. For exclusion due to no ACEs, the participant did not report on at least one instance of an ACE as assessed by the biobank (see Table 1).

Ethical approval for this study was provided by the University of Stirling General University Ethics Panel, Stirling, UK (reference: EC 2023 13946 9461); all research was performed in accordance with relevant guidelines/regulations.

### Variables

The UKB datasets include genetics, self-reported medical outcomes, mental health, and more. For this study, the UKB factors of interest examined for this analysis are displayed in Table 1. These UKB factors represent the variables of interest for this study, which were ACE history, self-reported anxiety, and a chronic pain condition for the primary outcomes. The secondary variable included the presence of the inflammatory biomarker CRP.

The independent variables were anxiety and history of childhood adversity, and the dependent variables were eight different pain measures (as listed in Table 1).

ACEs: Childhood adversity was operationalized by means of the five separate variables shown in Table 1 and scored as negative or positive (the third [item 20489] and fifth [item 20491] were reverse scored so that a higher score reflected more childhood adversity). Negative included reports of “felt hated”, “physically abused”, and/or “sexually abused” in childhood. Positive included “felt loved” and/or “taken to a doctor if needed”. NA reports were omitted.

Anxiety: Anxiety was operationalized as the three separate anxiety items (Table 1) scored as Yes/No for two of the items (i.e., items 20428, 20417) and by the reported frequency of the third (item 20515). In addition to yes and no responses, recoding was conducted to accommodate the N/A cases as “no”. This was because the first two items had ‘prefer not to answer’ and ‘do not know’ response options so we were confident handling missing or no response as reflecting some level of the respondent not agreeing with any of the options or simply that they were unwilling to identify themselves as having anxiety. This was felt to be the most conservative way to deal with missing data where we were unable to infer why a participant might choose not to answer a question. As per the UK Biobank’s study protocol (p18 and p69; available at https://www.ukbiobank.ac.uk/media/gnkeyh2q/study-rationale.pdf), it is also worth noting participants had the option to skip questions whether due to sensitivity or otherwise, and for privacy reasons this was deemed acceptable. Additionally, other recent studies have handled this similarly, such as Ramirez et al. (2022) in that they handled skipped UK Biobank questions by omitting them, and changing prefer not to answer to N/A since the true reason cannot be inferred retrospectively for participants^14^. For variables such as smoking, participants who skipped the questions were fully excluded.

Chronic pain: For pain, coding required at least one measure of chronic pain reported (Yes/No), and if available, the number of times pain was reported over the data collection period of the UKB (at the time of this analysis) was captured. As shown in Table 1, for all pain measures included in this analysis, duration was required to be at least 3 months to differentiate the pain as chronic versus acute. This set of reporting was defined as “Reports of pain”, which is a numeric variable counting and summing all the chronic pain reported (in any category from Table 1) across the average 7 years of follow-up currently available in UKB (representing a baseline Assessment Centre visit between 2006–2010, and follow-up visits 2012–2013, 2014 + , and/or 2019 +). This score represented all types of pain reported at each visit (from Table 1) across all years which were counted and aggregated into a single variable (most frequently ranging from 0–7). Pain data were collected at the Assessment Centre using a touchscreen self-report system for each visit.

C-reactive protein: CRP (item 30710) was measured by immunoturbidimetric-high sensitivity analysis on a Beckman Coulter AU5800 and captured over two assessments: the initial assessment between 2006 and 2010 (468,441 participants) and the first repeat assessment between 2012 and 2013 (17,835 participants). The UKB used CRP as a defined variable, meaning more than one instance may be present, and each instance represented a fixed identifiable set of results across all participants. For CRP, the defined-instances (visits) ran from 0 (baseline visit) to 1 (first follow-up visit) and were labelled using instancing 2 (the sum of 0–1 captured in mg/L). This data is considered ‘accruing’ and may have a comparable level at a later timepoint; to date we have instance 2 (the summary of visit 0 and 1). The UKB does note the presence of a dilution problem that was observed to increase with aliquot number for certain serum samples; however, as CRP has a high biological coefficient of variation, it was unaffected and did not require an estimation correction or adjustment. With the secondary analyses, the addition of CRP was captured by recorded visits and the CRP average (mg/L). N/A cases were omitted, and the mean and variance were calculated.

### Analyses

Coding for all analyses was conducted using R software^15^. As the dependent variable for the primary analysis (i.e., chronic pain) was count data, a Poisson regression model was determined to be the most suitable. This model enters all variables (i.e., the five ACE scores and the three anxiety indices) as well as their potential interactions. To answer the research questions, a generalized linear model using Poisson as the family (Poisson regression) was used. For questions with which the person could answer (yes, no, prefer not to answer), all the "yes" answers were coded as such, and any other answer was interpreted as a "no" (this includes non-answers). For the secondary CRP analysis, correlations between all variables of interest and CRP were assessed; Spearman correlations with Holm correction were used. Additionally for the CRP sample, a simplified version of the final model was created and compared against a corresponding adjusted version with socio-demographic/health behavior variables added in to identify how such factors may be affecting the results via a generalized linear mixed-effects model (GLM; with a Poisson distribution family).

Further details on the analysis decisions and background can be viewed in Appendix A. Additionally, Appendix B details the preliminary SEM conducted, which helped prioritize the analyses choices for the objectives. Stepwise regression was used to select the best fitting model (i.e., the combination of variables and interactions that better explain the dependent variable Reports of Pain). This puts all variables in and then iteratively remodels fit by backward elimination of non-significant variables (displayed in Table S2 of Appendix B). For missingness, the R na.omit() function was used to handle missing data as it removes any row that has a missing value in it. For large samples such as the UKB, this is standard practice.

### Ethical approval

Ethical approval for this study was provided by the University of Stirling General University Ethics Panel, Stirling, UK (reference: EC 2023 13946 9461).

### Generative AI statement

The author(s) did not use generative AI technologies for preparation of this work.

## Results

### Sample characteristics

The UKB demographics details can be reviewed in Table 2. Of the 9,238,453 men and women initially invited to join the UK Biobank, 503,317 (5.45%) provided informed consent and were recruited between 2006 and 2010. Overall, the participation rate was higher in women (participation rates were 6.4% and 5.1% in women and men, respectively)^16^.

**Table 2:** Self-reported ethnicity of UK biobank participants (recruited in 2006–2010) with census data for the age group 40–69 years in England, Wales, and Scotland in 2001 and 2011^a^.

Ethnicity^b^	UK Biobank (n = 499,877) No. of Persons	%	2001 UK Census (n = 20,198,307) No. of Persons	%	2011 UK Census (n = 23,146,612) No. of Persons	%
White^c^	472,837	94.6	19,085,322	94.5	21,133,317	91.3
Black or black British^d^	8066	1.6	302,073	1.5	565,777	2.4
Mixed^e^	2958	0.6	82,389	0.4	191,085	0.8
Indian	5951	1.2	325,651	1.6	442,338	1.9
Pakistani	1837	0.4	147,695	0.7	239,166	1.0
Bangladeshi	236	0.0	46,220	0.2	75,919	0.3
Chinese	1574	0.3	70,572	0.3	109,412	0.5
Other Asian	1858	0.4	73,917	0.4	240,324	1.0
Other ethnic group	4560	0.9	64,468	0.3	149,274	0.6

^a^Census datasets sourced from the Register Office and Office for National Statistics; National Records of Scotland; Northern Ireland Statistics and Research Agency^17^.

^b^Excludes 2,778 UK Biobank participants aged 40–69 years who were missing data on ethnicity or responded “prefer not to answer” or “do not know.”

^c^Included white British, white Irish, and other white backgrounds.

^d^Included Caribbean, African, and other black background.

^e^Included white and black Caribbean, white and black African, white and Asian, and other mixed ethnic backgrounds.

Each objective had a different sample size due to exclusion criteria and availability of records. Of the eligible cases, a total of 1077 records were omitted due to having missing responses in at least one of the variables of interest, and an additional 8 cases were omitted due to participants withdrawing from the UKB at the time of analysis. Thus, the final total number of cases available for the initial analysis was 24,164. In this sample, 61% reported female and 39% male. For the second objective, only n = 2007 individuals had records of CRP testing along with the initial inclusion criteria, so the sample size is considerably lower in comparison to the analysis for the first objective. In this second sample, 55% reported female and 45% male. For the simplified final CRP model to assess socioeconomics, five records were missing, making the total 2002 cases.

### Poisson regression results

#### Objective 1

The theoretical assumptions of the Poisson regression model were met, and the practical assumption of variance and mean being equals were also met (variance: 2.15, mean: 2.04). Stepwise regression was then used to select the best fitting model (i.e., the combination of variables and interactions that better explain the dependent variable [Reports of Pain]). The anxiety variable with the most influence on reported pain was the presence of muscular symptoms related to anxiety, followed by having informed a professional about their anxiety, and then the amount of trouble relaxing reported. With regard to ACE variables, the variable with the most influence was the frequency of physical abuse, followed by the frequency with which the person felt loved as a child. The remaining childhood trauma variables did not show a significant effect on reported pain. The full results are reported in Appendix Table S2.

#### Objective 2

**Figure 1:**
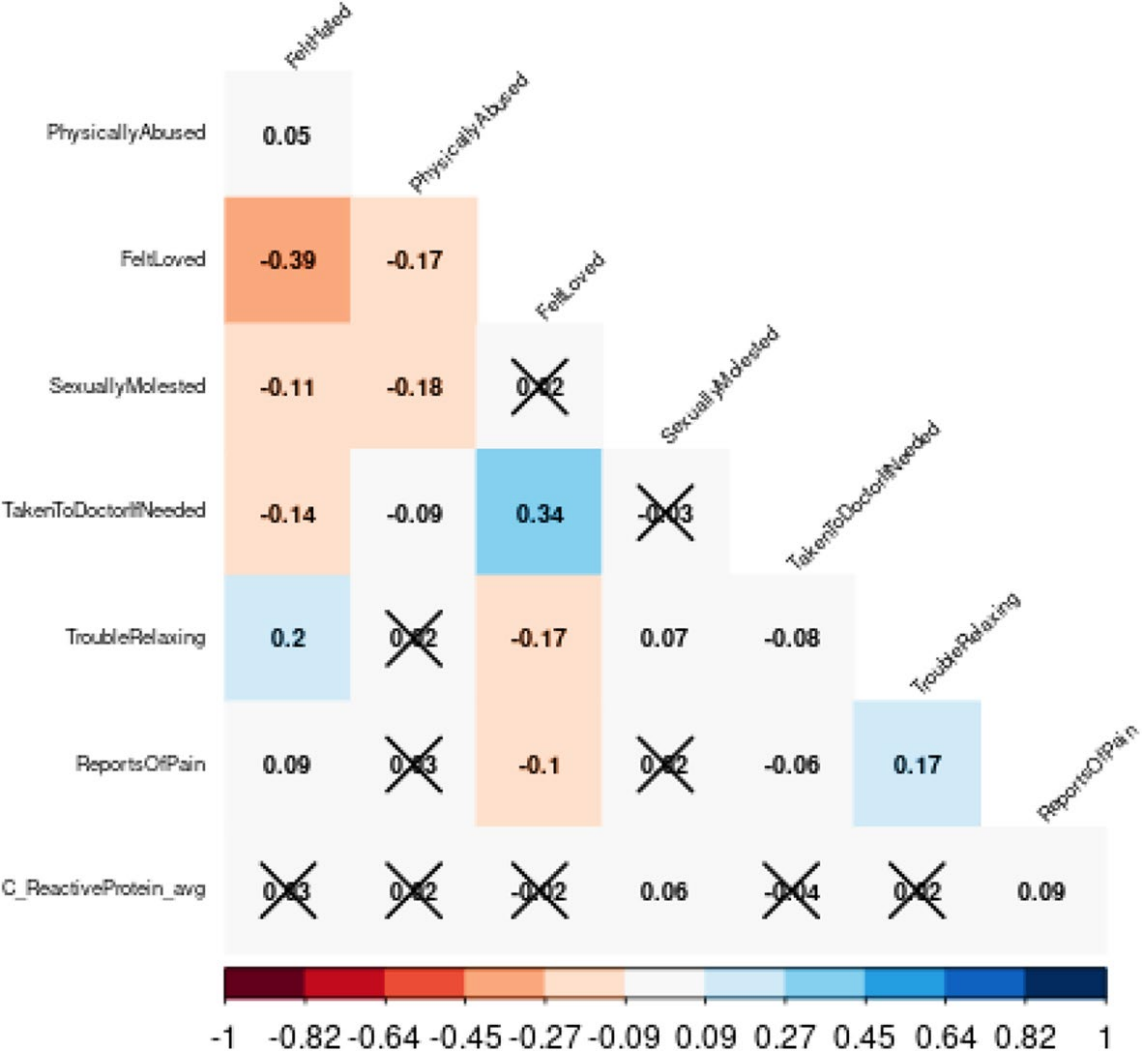
Heatmap of associations* between variables of interest and CRP. CRP, C-reactive protein. *X marks associations that were not statistically significant. In the shaded rows, each cell is shaded blue or red depending on the sign of the correlation, and with the intensity of color scaled 0–100% in proportion to the magnitude of the correlation (standard coding from red, [1, 0, 0], through white [1, 1, 1], to blue [0, 0, 1]. This bipolar scale of color leaves correlations near 0 empty (white) and makes positive and negative values of equal magnitude approximately equally intensely shaded.

All associations examined for the secondary analysis are displayed in the heat map correlation matrix (Fig. 1). In brief, the strongest correlation was between CRP and the number of times pain was reported over the years (*p* = 0.01), followed by CRP and the reported frequency of sexual abuse in childhood (*p* = 0.05).

Additionally, Poisson regression models were conducted with the smaller sample for which CRP levels were available to assess the potential pathways of any relationships between the three concepts of interest and chronic pain as the outcome variable. These results are shown in Table 3. Of note, there was an important interaction between informing a professional about anxiety and CRP in predicting pain. Childhood abuse also interacts significantly with CRP to predict pain.

**Table 3:** Poisson model: secondary objective (with CRP as the predictor of chronic pain).

Variable	Estimate	Std. error	z-value	*P*-value
(Constant)	0.807	0.093	8.659	< 2e−16
Felt Hated as a child (Likert)	0.071	0.033	2.158	**0.031**
Physically Abused as a child (Likert)	0.100	0.023	4.351	** < 0.001**
Sexually Abused as a child (Likert)	0.118	0.045	2.621	**0.009**
Felt Loved as a child (Likert)	− 0.011	0.017	− 0.630	0.529
Taken to Doctor if Needed as a child (Likert)	− 0.040	0.017	− 2.320	**0.020**
Has informed a professional about anxiety episodes				
No (ref)				
Yes	0.030	0.058	0.520	0.603
Trouble relaxing during anxiety episodes (Likert)	0.134	0.034	3.991	** < 0.001**
Muscle symptoms during anxiety episodes				
No (ref)				
Yes	0.239	0.059	4.025	** < 0.001**
CRP average levels	0.011	0.020	0.547	0.584
Interactions				
Trouble relaxing X sexually abused	− 0.014	0.019	− 0.733	0.464
Physically abused X sexually abused	− 0.080	0.019	− 4.326	** < 0.001**
Muscle symptoms X physically abused	− 0.089	0.042	− 2.127	**0.033**
Has informed a professional about anxiety X felt hated as a child	0.000	0.034	0.010	0.992
Trouble Relaxing X felt hated as a child	− 0.013	0.017	− 0.773	0.439
Has informed a professional about anxiety X CRP	0.036	0.014	2.679	**0.007**
Trouble relaxing X CRP	− 0.009	0.008	− 1.133	0.257
Muscle symptoms X CRP	0.005	0.012	0.391	0.696
Felt hated X CRP	− 0.003	0.009	− 0.393	0.694
Physically abused X CRP	− 0.017	0.005	− 3.324	**0.001**
Sexually abused X CRP	− 0.023	0.011	− 2.113	**0.035**
Felt loved X CRP	− 0.003	0.004	− 0.608	0.543
Taken to doctor if needed X CRP	0.009	0.004	2.404	**0.016**
Trouble relaxing x sexually abused X CRP	0.002	0.004	0.577	0.564
Physically abused x sexually abused X CRP	0.019	0.005	3.970	** < 0.001**
Muscle symptoms x physically abused X CRP	0.013	0.010	1.348	0.178
Has informed a professional about anxiety X felt hated X CRP	− 0.018	0.008	− 2.150	**0.032**
Trouble relaxing X felt hated X CRP	0.003	0.004	0.636	0.525

Analysis notes: dependent variable: number of instances of pain reported through the years. N = 2007. Null deviance: 3022.5 on 2006 degrees of freedom. Residual deviance: 2764.4 on 1979 degrees of freedom. AIC: 8351. Significant values are in bold.

To further break down, contextualize, and examine the results of the Poisson regression more clearly, variable regression plots were conducted. These studies investigated each individual relationship, isolating it and plotting what it looked like when all the other variables were held constant. All significant effects and interactions pertaining to CRP were included, and the results are categorized as interactions and 3-way interactions. Individual effects of anxiety and ACEs were also assessed via partial regression plots (Appendix Figure S4).

#### Interactions with CRP

**Figure 2:**
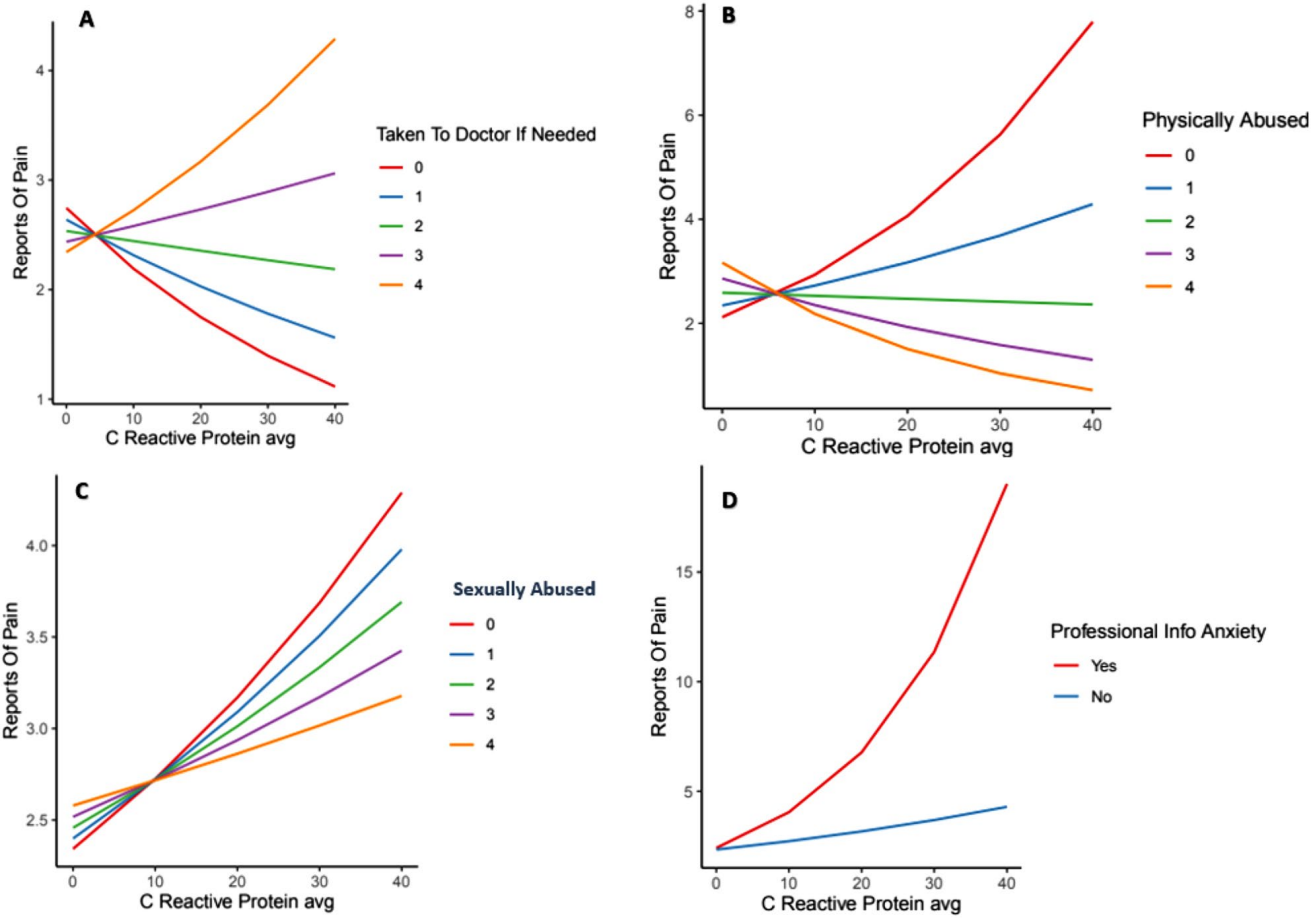
(**A**) Interaction between being taken to the doctor when needed as a child and CRP as predictors of chronic pain, (**B**) Interaction between experiencing physical abuse as a child and CRP levels as predictors of chronic pain, (**C**) Interaction between experiencing sexual abuse as a child and CRP as predictors of chronic pain, (**D**) Interactions between having informed a professional about anxiety x CRP. *Note*: 0–4 scale = never–very often true.

Interestingly, CRP was a stronger predictor for patients who reported never having experienced physical abuse (Fig. 2B). The more frequently physical abuse was experienced or reported, the weaker the relationship between chronic pain and CRP became, eventually undergoing an inversion. Similar to the interaction results of physical abuse, CRP was a stronger predictor for patients who were not exposed to sexual abuse as children (Fig. 2C). As the frequency of sexual abuse increased, CRP levels appeared to become less of a deciding factor.

There was a substantial divide in the effect of CRP on chronic pain, as displayed in Fig. 2A, depending on the availability of doctor visits during childhood. For patients who had low or infrequent availability to visit the doctor when needed as children, higher CRP levels tended to decrease chronic pain reported during adulthood. In contrast, patients who had a high availability of visiting the doctor when needed showed CRP levels to be a strong predictor of chronic pain in adulthood.

As indicated by Fig. 2D, CRP played a significant role in predicting chronic pain for patients with anxiety (i.e., those who had sought professional help). In comparison, for patients without anxiety or whose anxiety had not driven them to discuss with a professional, CRP gradually lost significance and became a very weak predictor of chronic pain.

#### Three-way interactions

**Figure 3:**
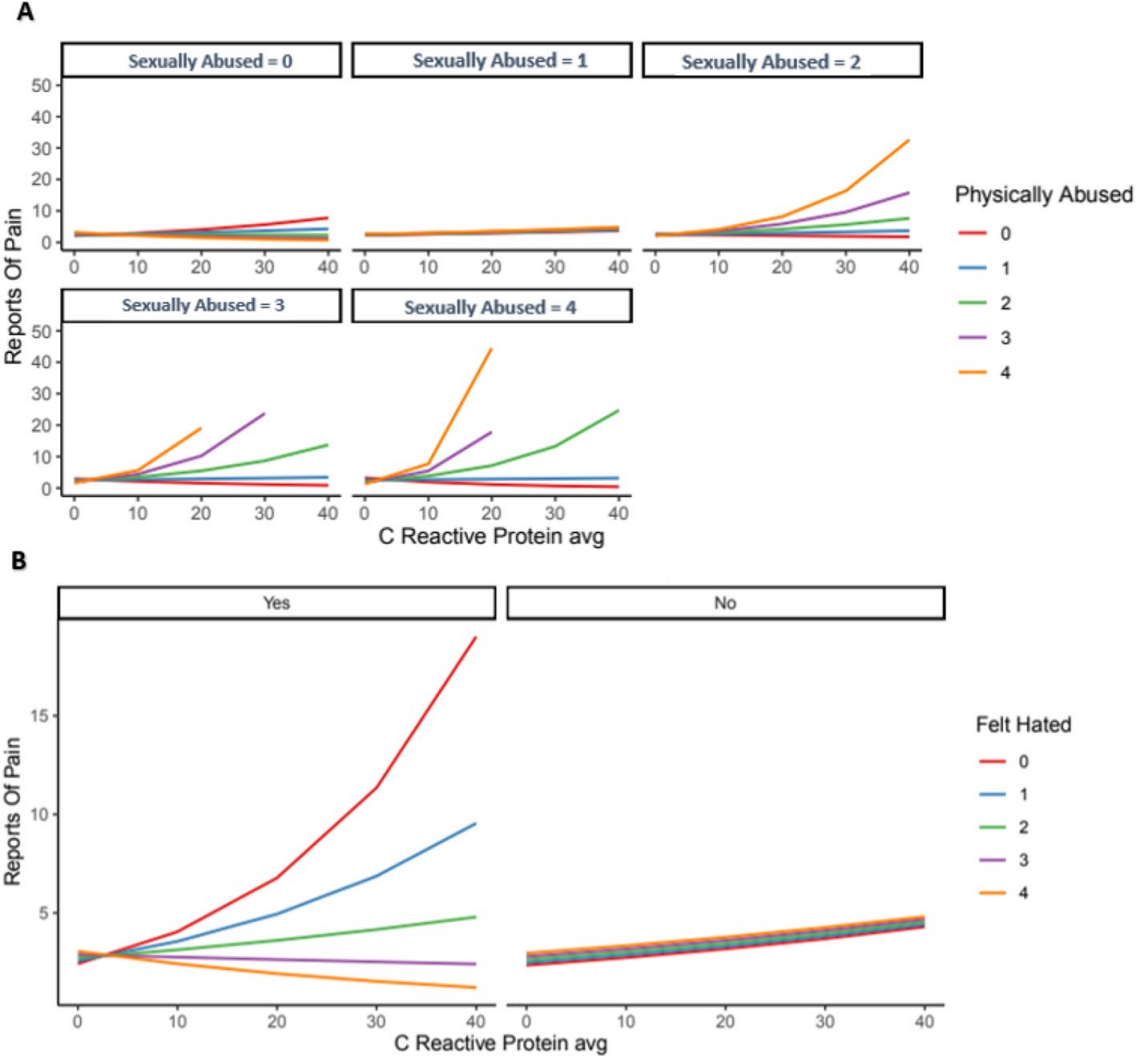
3-way interactions between: (**A**) CRP, sexual abuse, and physical abuse during childhood, (**B**) CRP, reporting anxiety to a professional x feeling hated as a child. *Note*: 0–4 scale = never–very often true.

As shown in Fig. 3, when both sexual abuse and physical abuse were present in childhood, they greatly potentiated the effect that CRP levels had on chronic pain as a predictor (Fig. 3A). For the final examination, patients who felt hated more frequently as a child were less sensitive to CRP levels as a predictor of chronic pain (Fig. 3B). This interaction, however, only applied to patients who had reported "yes” to having discussed anxiety with a professional. For patients who had reported “no” for having discussed anxiety with a professional, this interaction disappeared.

#### Simple socio-demographic adjusted analysis

Four socioeconomic variables which had sufficient coverage were available in the UKB dataset: sex (male/female), ever smoked (yes/no), alcohol use status (current, past, never), and age at recruitment (range 40–70 years). The generalized linear mixed-effects model results and corresponding simplified final model were built and compared to identify if the available data on socioeconomic factors affected the model results (see Appendix C for expanded details on the methodology). The linear mixed-effects model included age at recruitment as a random intercept, and the inclusion of a random slope (allowing the variable’s slope to vary with age at recruitment) for each dependent variable was tested. ANOVAs comparing the model with the random slope versus without for a given variable indicated that including a random slope was significant at the 95% level for ProfessionalInfoAnxiety (χ^2^(2) = 28.439, *p* < 0.001), MuscleSymptomsAnxiety (χ^2^(2) = 8.0571, *p* = 0.018) and PhysicallyAbused (χ^2^(2) = 17.409, *p* < 0.001), and thus these were included in the mixed-effects model. Other than the inclusion of socioeconomic control variables for sex, ever smoked, alcohol use, and age at recruitment as a random effect (with the given random slopes) for the mixed-effects model, the models were identical. Although the mixed effects model had a slightly better fit (AIC 8275 for the mixed effects versus AIC for the simplified final model), the coefficient estimates for all variables included in both models was very similar, and almost all variables which had significant coefficients (*p* < 0.05) in the simplified final model also had significant coefficients in the mixed-effects model (with the exception of TakenToDoctorIfNeeded x C_ReactiveProtein_avg). The mixed effects control variables ever smoked, sex, and alcohol drinker were not significant (*p* > 0.05); age at recruitment as a random effect was not assigned a significance (see Table S4 for variances and standard deviations). In this case, although the inclusion of the available socioeconomic factors in the mixed-effects model improved the fit on the data, it did not change the conclusions regarding the predictors of the number of times pain was reported.

## Discussion

The objective of this study was to examine the relationships between reported ACEs, anxiety, and chronic pain and to assess the associations between ACEs, anxiety, and CRP levels, as well as the link between CRP and chronic pain. Exploring these associations in more detail, using a Poisson regression, demonstrated some interesting main effects with three significant interactions identified in explaining chronic pain experiences. For anxiety, an increased frequency of having trouble relaxing during anxiety episodes and experiencing muscle symptoms was associated with an increase in reported chronic pain, and there was an important interaction between informing a professional about anxiety and CRP in predicting pain. With respect to ACEs, patients who reported feeling hated more frequently as a child, as well as patients who were physically or sexually abused as a child, reported more chronic pain in adulthood. With respect to the interaction analyses, the most influential interaction was between the frequency of physical abuse experienced as a child and reported muscular symptoms during anxiety. The other significant interactions were between the frequency with which they felt hated as a child and having discussed anxiety with a professional and between the reported frequency of sexual abuse in childhood and difficulties relaxing during anxiety attacks.

Some of the associations between ACEs (as were shown in Fig. 1) appear at odds with the prevailing literature, for example, sexual abuse being inversely associated with physical abuse. This may reflect variations between the present study and others in ACE definitions or study sample characteristics. Recent research by Broekhof et al. (2022) suggests that due to overlap between ACE sub-types and individual ACEs, perhaps ACEs should be assessed as a combined group rather than individually^18^.

The findings further expand on previous research, such as multivariate analysis^19^, which concluded that any ACE measure was associated with a higher risk of both anxiety and depression. Children exposed to four ACEs or more had higher odds of anxiety and depression, for example, than those exposed to fewer than four ACEs. The results of the present study agree with this previous analysis but also support the assessment of the impact of ACEs on internalizing behaviors separately instead of grouping anxiety and depression outcomes together. The present results are also interesting to consider in light of a recent study^20^ that showed that increased or chronic exposure to ACEs are key contributing drivers to chronic pain earlier in a person’s life. This study by Groenewald also demonstrated that exposure to one or more ACEs was associated with a 60%–170% increase in the likelihood of experiencing chronic pain. Taken together, it seems that the frequency of ACE exposure may be tied to chronic pain prevalence in adults.

Interestingly, addressing the second objective provided more insight into the underlying mechanisms of the associations. CRP was found to be a stronger predictor of pain for patients who reported never having experienced physical abuse, meaning that the more frequently physical abuse was reported, the weaker the relationship between chronic pain and CRP became. Similarly, CRP was a stronger predictor for patients who were not exposed to sexual abuse as children, showing that as the frequency of sexual abuse increased, CRP levels appeared to become less of a predictor. The interaction results did show a substantial divide in the effect of CRP on chronic pain. For patients who had low or infrequent availability to visit the doctor when needed as children, higher CRP levels were related to a decrease in the frequency of chronic pain reported during adulthood. In contrast, patients who had a high availability of visiting the doctor when needed showed CRP levels to be a strong predictor of chronic pain in adulthood. In addition to CRP, systemic inflammation related to elevated levels of inflammatory biomarkers such as TNF-Α, IL-6, and IL-1B has previously been linked to increased inflammatory and neuropathic pain^21^. The role of inflammation in the pathogenesis of other health problems in adults may be one of the main psychobiological mechanisms underlying the relationship between ACE history and poor health outcomes. In a study by lob et al.^22^ assessing CRP and hair cortisol, adults with three or more ACEs had a higher risk of elevated CRP levels across a 4-year period. Such results demonstrate how the complexities between ACEs and proinflammatory responses may persist and impact later stages of adulthood and should be factored into chronic pain screening as well. In addition, the number and specific combination of ACE types may influence CRP levels, as suggested by our contrasting individual interaction findings.

For the 3-way interactions, while CRP had a significant role in predicting chronic pain for patients with reported anxiety, this effect was much weaker for patients without anxiety. Of note, although both sexual abuse and physical abuse during childhood each decreased the effect CRP levels had on predicting chronic pain when examined individually for their interactions, when sexual abuse and physical abuse were both present, they significantly increased the effect that CRP levels had on predicting chronic pain in adulthood. This may be because those with a history of multiple traumatic experiences have higher inflammation. Prior meta-analyses of cross-sectional studies have confirmed the association of inflammation with traumatic experiences, and longitudinal studies have provided evidence supporting a bidirectional association that elevated inflammation may contribute to trauma symptoms and that trauma symptoms contribute to elevated inflammation^9^.

In terms of implications for future treatment, there is some support for additive therapy with anti-inflammatory medication for treatment-resistant depression; however, this was only true for patients with low-level inflammation (CRP ≥ 3 mg/L)^23–25^. It may be worth examining the potential for anti-inflammatory medications for treating patients with anxiety, particularly those with elevated inflammatory markers such as CRP. It would be worth investigating a cohort of patients with anxiety who could potentially benefit from individualized therapy with anti-inflammatory drugs, such as those with chronic pain and a history of ACEs.

While interesting, the cross-sectional nature of these data suggests caution in making any definitive statements about directionality of effects. For example, pain (a potent stressor) might lead to elevated CRP levels just as much as elevated CRP could lead to heightened pain sensitivity. Future studies should consider other variables that could influence CRP levels that might confound the associations between CRP and pain (e.g., demographics, socioeconomic status, health behaviors, social isolation etc.) and exploring the impact of these factors on the analyses reported here could shed light on variables which modify how ACEs relate to chronic pain. Although more research into the relationships is needed, the results of the present study are meaningful in that they highlight that elevated CRP is potentially associated not only with childhood trauma history and adult chronic pain outcomes but also with anxiety symptomology. This suggests that it may be important to consider the underlying inflammatory component potentially present in this specific adult population (ACE history, anxiety, and chronic pain).

### Limitations

The dataset used was a substantial sample size; however, some of the data generated included online questionnaires that relied on self-report. While such an approach was necessary to collect information from a larger sample, this may have affected the quality of the data due to recall bias. However, the only alternative to surveys (clinical interviews) for abuse and anxiety measures also relies on self-report and recall and would not be pragmatic in epidemiological research. Although appropriate statistical methods were used, findings related to biomarker data should be interpreted cautiously. These findings could still be affected by unmeasured or unaccounted for confounding variables. However, multiple sensitivity analyses were conducted, and the significant results appear to not be affected by biases. Finally, the dataset was primarily based in the UK, so it may not be globally generalizable across countries and cultures. However, the sample size itself was quite robust and opens opportunities for cross-cultural comparison with other large datasets.

## Conclusion

The results of this analysis are meaningful in that they indicate that CRP is significantly associated not only with certain ACEs and adult chronic pain outcomes but also with anxiety symptoms. Of note, childhood abuse significantly interacted with CRP to predict pain, and there were important results on the combined effect of specific ACEs. The implications of this connection warrant further study to validate these relationships and potential mediations but are an important consideration in the underlying inflammatory components of systemic dysfunction after childhood adversity in adults with chronic pain, anxiety, and higher levels of CRP.

### Supplementary Information

Supplementary Information.

## Data Availability

This research was conducted using data from the UK Biobank, a major biomedical database that can be accessed at www.ukbiobank.ac.uk. The data that support the findings of this study are available from the corresponding author upon reasonable request.
