# Supplementary material for: An analysis on history of childhood adversity, anxiety, and chronic pain in adulthood and the influence of inflammatory biomarker C-reactive protein
Source: Sci Rep. 2023 Oct 21;13:18000. doi: 10.1038/s41598-023-44874-1 (PMC10590370; doi:10.1038/s41598-023-44874-1)
Supplement: Supplementary file 1 — Supplementary Information. [file 41598_2023_44874_MOESM1_ESM.docx]

**Appendix A. Additional statistical background**

A two-way ANOVA was initially conducted to accommodate multiple independent variables. The independent variables were anxiety and history of childhood adversity, and the dependent variables were eight different pain measures (as listed in **Table S1**). The assumption of normality of residuals was violated, so ultimately, ANOVA was not recommended as the best model for this study. For transparency, the results of this failed ANOVA are displayed in **Table S1.**

**Table S1. Results of failed ANOVA testing**

|  | **Df** | **SS** | **MS** | **F** | ***P*** | **eta-squared** | **partial**  **eta-squared** |
| --- | --- | --- | --- | --- | --- | --- | --- |
| ProfessionalInfoAnxiety | 1 | 632 | 632.2 | 303.892 | < 0.001 | 0.0005 | 0.0005 |
| TroubleRelaxing | 1 | 535 | 534.8 | 257.082 | < 0.001 | 0.0045 | 0.0047 |
| MuscleSymptomsAnxiety | 1 | 445 | 444.9 | 213.865 | < 0.001 | 0.0076 | 0.0078 |
| FeltHated | 1 | 99 | 99.4 | 47.77 | < 0.001 | 0.0009 | 0.0009 |
| PhysicallyAbused | 1 | 37 | 36.9 | 17.719 | < 0.001 | 0.0006 | 0.0006 |
| SexuallyAbused | 1 | 35 | 34.6 | 16.64 | < 0.001 | 0.0006 | 0.0006 |
| FeltLoved | 1 | 13 | 12.6 | 6.05 | < 0.001 | 0.0002 | 0.0002 |
| TakenToDoctorIfNeeded | 1 | 2 | 1.6 | 0.749 | < 0.001 | 0.0000 | 0.0000 |
| Residuals | 24163 5 | 268 | 2.1 |  |  |  |  |

*Regression analysis background:*

After determining ANOVA was an inappropriate model, it was assessed that Poisson regression made the best fit due to the count data of the UKB. Count data are discrete and left-censored at zero (that is, counts usually cannot be less than zero). Count data are often very skewed and produce skewed residuals if a parametric approach is attempted.

In ordinary least square (OLS) regression, the R2 statistic measures the amount of variance explained by the regression model. The value of R2 ranges in [0,1], with a larger value indicating that more variance is explained by the model (a higher value is better).


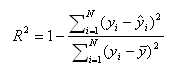


*N* is the number of observations in the model, *y* is the dependent variable, *y*-bar is the mean of the *y* values, and *y*-hat is the value predicted by the model. The numerator of the ratio is the sum of the squared differences between the actual *y* values and the predicted *y* values. The denominator of the ratio is the sum of squared differences between the actual *y* values and their mean.

The three main ways to interpret R2 are as follows.

- explained variable: how much variability is explained by the model
- goodness-of-fit: how well the model fits the data
- correlation: the correlations between the predictions and true values

For logistic regression, there have been many proposed pseudo-R2. A nonexhaustive list is shown below.

- Efron’s R2
- McFadden’s R2
- McFadden’s Adjusted R2
- Cox & Snell R2
- Nagelkerke/Cragg & Uhler’s R2
- McKelvey & Zavoina R2
- Count R2
- Adjusted Count R2

For this study, Cragg & Uhler’s was conducted:

**
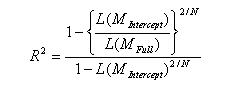
**

Nagelkerke or Cragg & Uhler’s adjusts Cox & Snell’s approach so that the range of possible values extends to 1. To achieve this, the Cox & Snell R-squared is divided by its maximum possible value, 1-*L(M_Intercept_)^2/N^.* Then, if the full model perfectly predicts the outcome and has a likelihood of 1, Nagelkerke/Cragg & Uhler’s R-squared = 1. When *L(M_full_) = 1*, then *R^2^ = 1*; when *L(M_full_) = L(M_intercept_)*, then *R^2^ = 0*.

When analyzing data with a logistic regression, an equivalent statistic to R-squared does not exist. The model estimates from a logistic regression are maximum likelihood estimates arrived at through an iterative process. They are not calculated to minimize variance, so the OLS approach to goodness-of-fit does not apply. However, to evaluate the goodness-of-fit of logistic models, several pseudo R-squareds have been developed.

The interpretation of an OLS R-squared is relatively straightforward: “the proportion of the total variability of the outcome that is accounted for by the model”. In building a model, the aim is usually to predict variability. The outcome variable has a range of values, and you are interested in knowing what circumstances correspond to what parts of the range [25].

**Appendix B. Additional results**

Stepwise regression was used to select the best fitting model (i.e., the combination of variables and interactions that better explain the dependent variable (Reports of Pain). This is displayed in **Table S2**.

**Table S2. Poisson regression models: primary objective**

| **Variable** | **β** | **95% CI** | | **Std. Error** | **z value** | ***P*-value** |
| --- | --- | --- | --- | --- | --- | --- |
|  |  | **Low** | **Upper** |  |  |  |
| **(Intercept)** | 0.5612 | 0.5141 | 0.6083 | 0.0240 | 23.361 | < 0.001 |
| **ProfessionalInfoAnxietyYes** | 0.0604 | 0.0312 | 0.0894 | 0.0148 | 4.066 | < 0.001 |
| **TroubleRelaxing** | 0.0424 | 0.0266 | 0.0581 | 0.0081 | 5.257 | < 0.001 |
| **MuscleSymptomsAnxietyYes** | 0.1937 | 0.1626 | 0.2246 | 0.0158 | 12.266 | < 0.001 |
| **FeltHated** | 0.0157 | -0.0003 | 0.0318 | 0.0082 | 1.915 | 0.055 |
| **PhysicallyAbused** | 0.0300 | 0.0180 | 0.0420 | 0.0061 | 4.912 | < 0.001 |
| **SexuallyAbused** | 0.0076 | -0.0137 | 0.0289 | 0.0109 | 0.702 | 0.483 |
| **FeltLoved** | -0.0101 | -0.0192 | -0.0011 | 0.0046 | -2.196 | 0.028 |
| **TakenToDoctorIfNeeded** | -0.0039 | -0.0127 | 0.0049 | 0.0045 | -0.871 | 0.384 |
| **TroubleRelaxing X SexuallyAbused** | 0.0107 | 0.0012 | 0.0201 | 0.0048 | 2.226 | 0.026 |
| **PhysicallyAbused X SexuallyAbused** | -0.0076 | -0.0157 | 0.0004 | 0.0041 | -1.863 | 0.063 |
| **MuscleSymptomsAnxietyYes X PhysicallyAbused** | -0.0260 | -0.0452 | -0.0069 | 0.0098 | -2.668 | 0.008 |
| **ProfessionalInfoAnxietyYes X FeltHated** | -0.0176 | -0.0337 | -0.0015 | 0.0082 | -2.137 | 0.032 |
| **TroubleRelaxing X FeltHated** | 0.0067 | -0.0011 | 0.0144 | 0.0039 | 1.687 | 0.092 |

Null deviance: 20064 on 24163 degrees of freedom. Residual deviance: 19199 on 24150 degrees of freedom. AIC: 78978.

Since our dependent variable was count data, a Poisson model was determined to be the most suitable, and the practical assumption of variance and mean being equals were also met (variance: 2.15; mean: 2.04). The model significantly predicted the number of times pain was reported through the years (χ²(13) = 864.96, p < 0.001, pseudo-R² (Cragg-Uhler) = 0.04).

*SEM results*

The exploratory factor analysis (EFA) results of the Kaiser-Meyer-Olkin (KMO) index calculation are shown in **Table S3**. A minimum KMO index of 0.5 was needed for EFA, and since all the variables were above the 0.5 threshold, it was determined that the EFA was adequate.

**Table S3. Measure of sampling adequacy (MSA) for each variable**

| **MuscleSymptomsAnxiety** | **ProfessionalInfoAnxiety** | **FeltHated** |
| --- | --- | --- |
| 0.63 | 0.64 | 0.66 |
| **PhysicallyAbused** | **FeltLoved** | **SexuallyAbused** |
| 0.71 | 0.63 | 0.69 |
| **TakenToDoctorIfNeeded** | **TroubleRelaxing** | **ReportsOfPain** |
| 0.62 | 0.79 | 0.80 |
| **C_ReactiveProtein_avg** |  |  |
| 0.72 |  |  |

Kaiser-Meyer-Olkin factor adequacy. Call: KMO (r = proteinDF_numerics). Overall Measure of Sampling Adequacy = 0.67.

The second-step results of the parallel analysis suggested that the number of factors was four and the number of components was two (shown in **Figure S1**). After examining the results, it was determined that a good starting point would be exploring three factors, and the PCA approach was used since it produced higher eigenvalues for the first three factors/components.


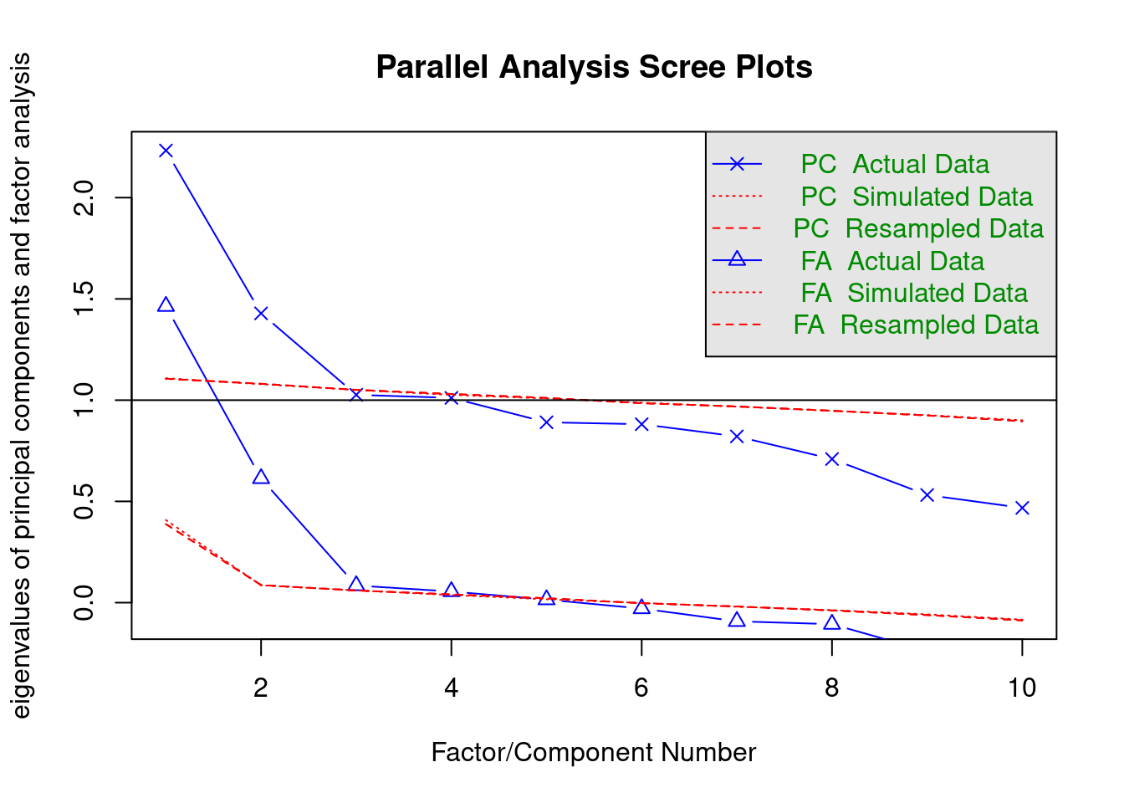


**Figure S1. Parallel Analysis Scree Plots**

FA, factor analysis; PC, principal component.

Performing a hierarchical cluster analysis also indicated that a 3-factor structure was a sensible solution, as demonstrated in **Figure S2**.


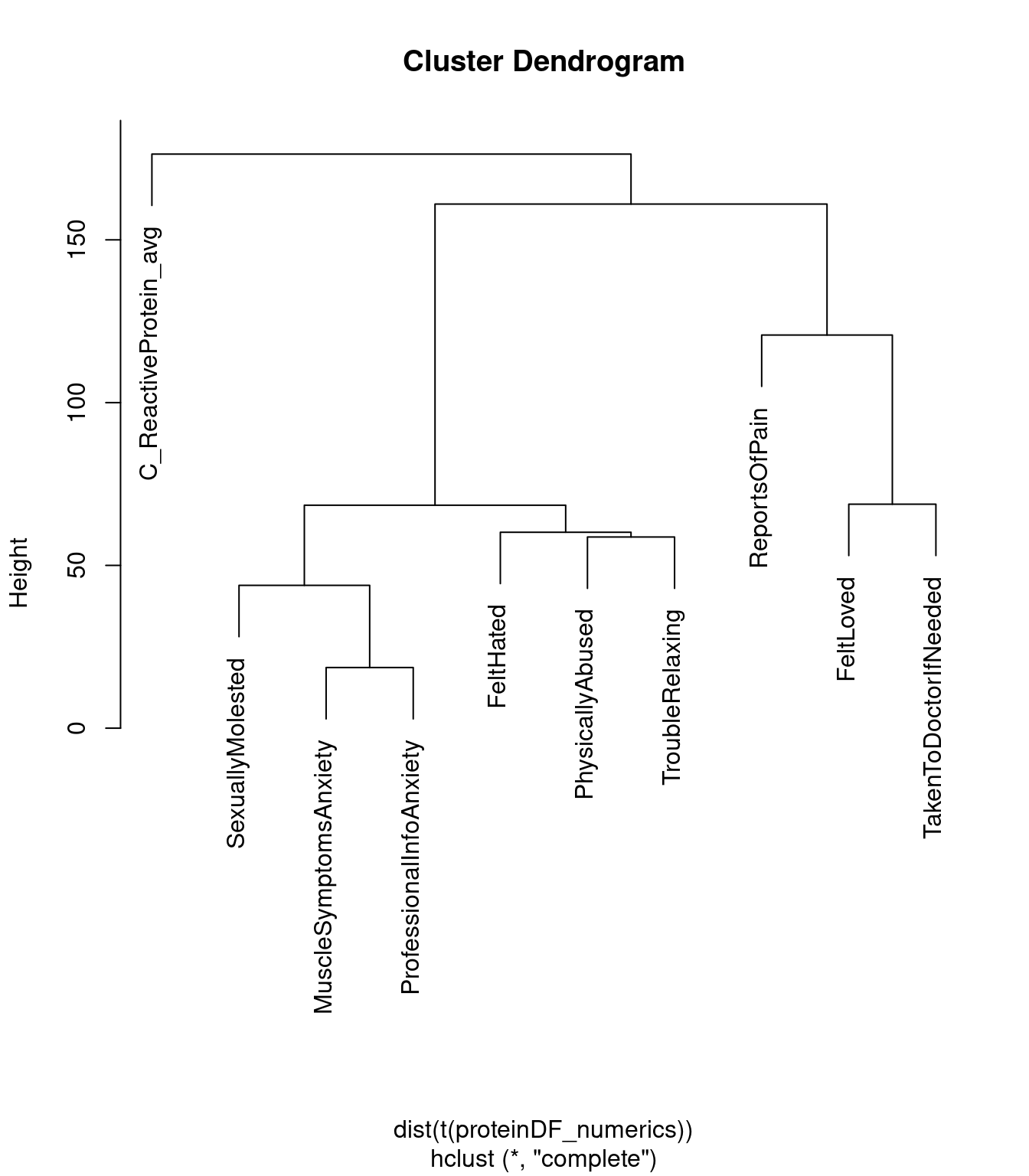


**Figure S2. Cluster Dendrogram**

The results of the factor analysis with three factors showed a mean item complexity = 1.3 (the test of the hypothesis that three components were sufficient). The root mean square of the residuals (RMSR) was 0.11 with an empirical chi-square of 2362.57 (probability < 0). The fit based upon the off-diagonal values was 0.5.


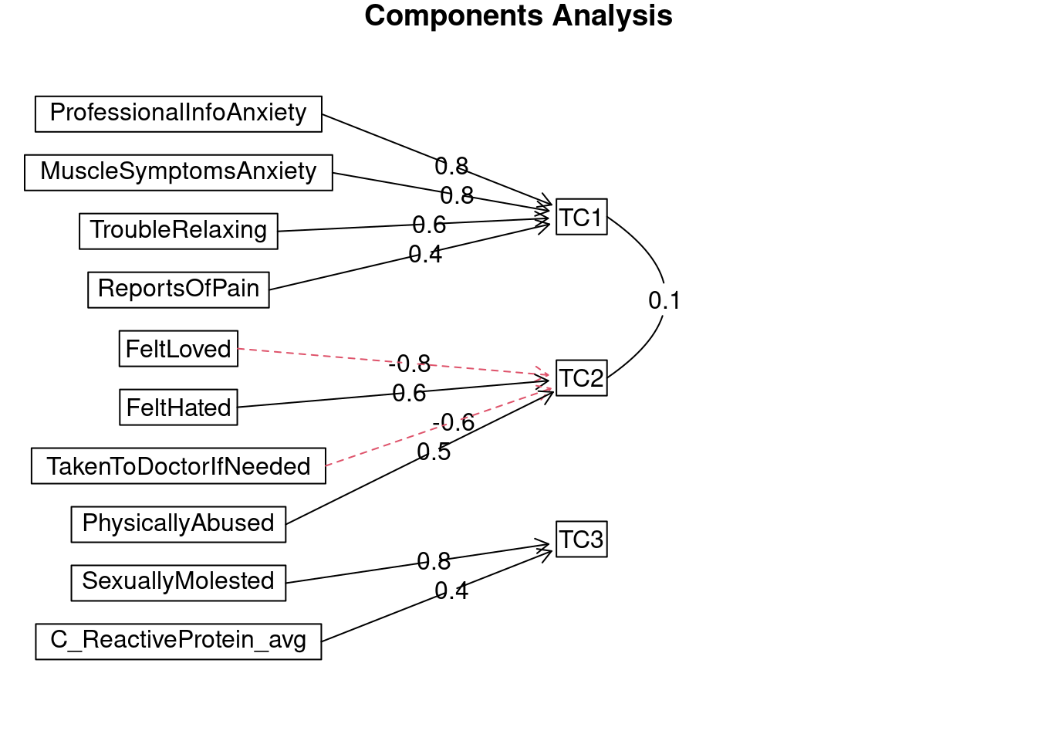


**Figure S3. Component analysis**

Bold-line arrows represent main factor loadings, and dashed red lines represent cross-loadings.

*SEM for chronic pain and relevant UKB variables*

Based on the visualizations in the component analysis (**Figure S3**), the variables tended to show three main groups: one related to anxiety, another related to childhood adversity, and the final one containing CRP in isolation along with history of sexual abuse. Some variables were ultimately excluded to improve the goodness of fit. In particular, discussing anxiety with a professional and sexual abuse experience both decreased the fit considerably. Our assumption in the case of sexual abuse is that it is not a commonly reported experience in databases available for CRP levels, so the limited experiences may have dominated over the rest of the variables. For discussing anxiety with a professional, it is not clear why including it reduced the fit, but it may be worth exploring in future modeling. The final best fit achieved is shown in the following diagram (**Figure S4**).


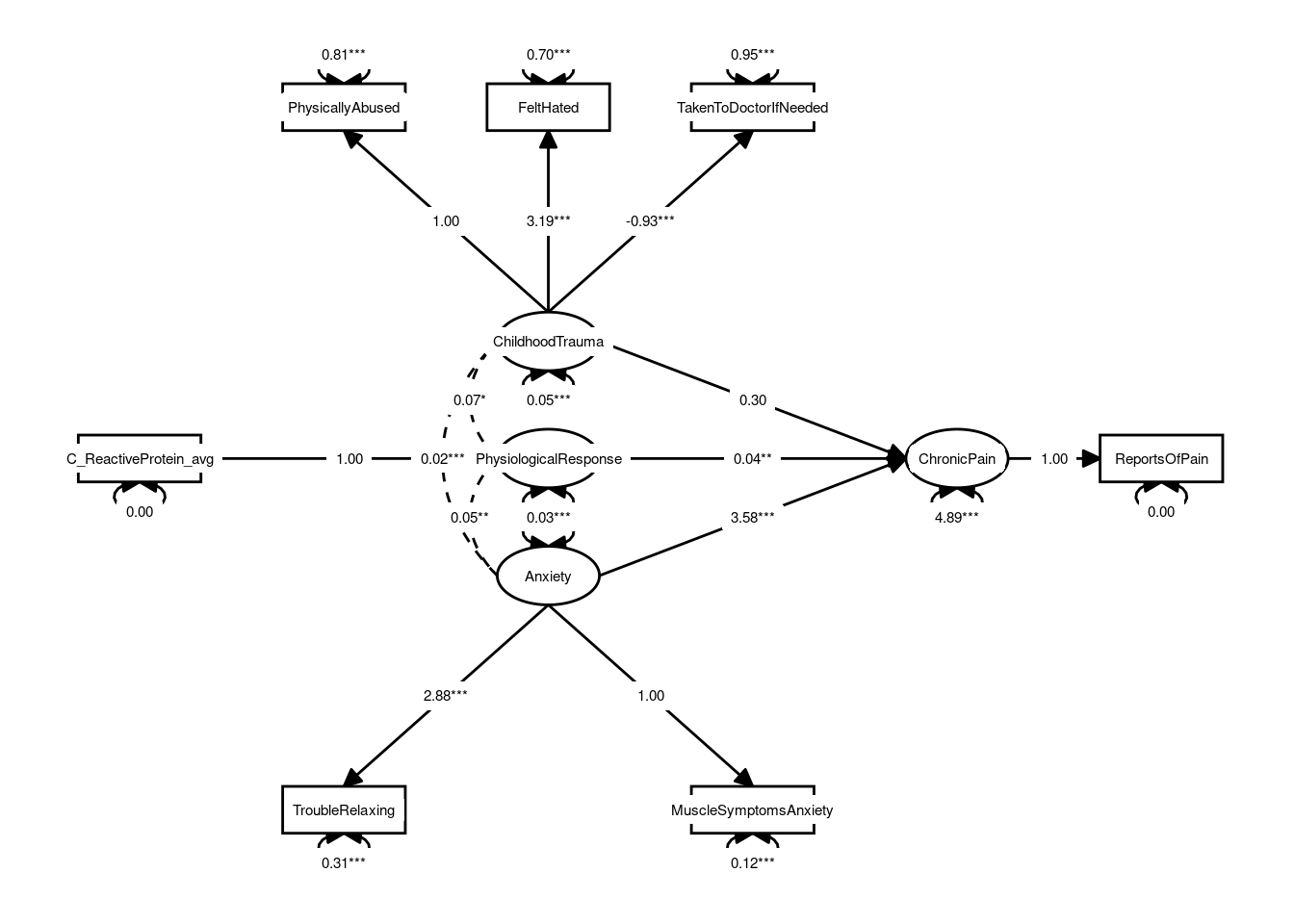


**Figure S4. Diagram illustrating the best fit for the SEM**

This model has the following fit parameters: (in parentheses are the necessary values for a good fit) CFI: 0.975 (> 0.90), TFI: 0.948 (> 0.90), RMSE: 0.026 (< 0.05), **χ**² / *df* = 2.36 (< 3).

This model considered subjective reports of anxiety as measurements of anxiety, modeled as a latent construct. Anxiety had a strong association with chronic pain. It could be suggested that CRP levels are part of a larger physiochemical response that is associated with chronic pain. Childhood trauma is modeled as measured by physical abuse, feeling hated as a child, and lack of availability of medical care when needed during childhood. Additionally, in this model, chronic pain was a latent variable measured only by the number of reports of pain over the years. Based on this, anxiety, childhood abuse, and CRP were indeed predictors of chronic pain. Childhood abuse, however, does not predict it very well when compared with the other two variables. Anxiety was by far the best predictor of chronic pain according to the SEM.

*Individual effects of anxiety and ACEs*

As shown in **Figure S5**, patients who reported feeling hated more frequently as a child reported more chronic pain in adulthood (**part D**). The frequency of physical abuse suffered as a child had an influence on chronic pain during adulthood, increasing how often chronic pain was reported (**part C**). Patients who reported being sexually abused more frequently as children had a slightly increased number of chronic pain experiences reported (**part A**). Patients who reported being taken to the doctor more frequently as children displayed slightly fewer reports of chronic pain during adulthood (**part B**). An increased report of having trouble relaxing during anxiety episodes was associated with an increase in the reported frequency of chronic pain (**part E**). Patients who experienced muscle symptoms during anxiety episodes reported more chronic pain than those who did not experience muscular symptoms with anxiety (**part F**). These partial regression plots show the estimated relationship between the response and an explanatory variable after accounting for the other variables in the model (the bold line/dots show the association between variables; the shaded area represents confidence or the uncertainty around the functional estimate).


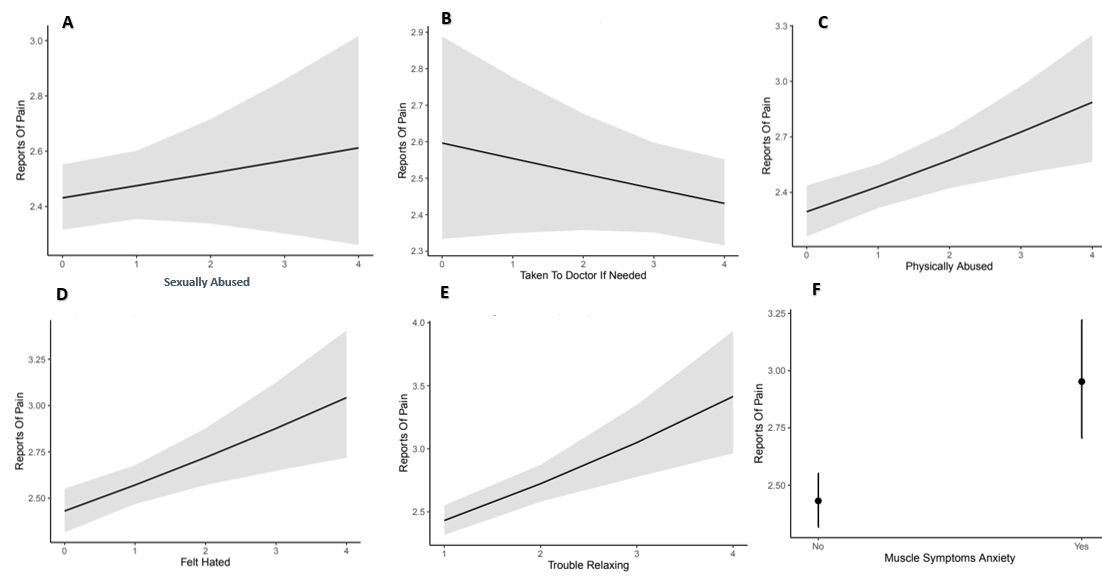


**Figure S5.** A) Partial regression plot on the association between sexual abuse frequency x chronic pain, B) Association between being taken to the doctor when needed as a child x chronic pain, C) Association between physical abuse in childhood and chronic pain in adulthood, D) Association between reports of feeling hated during childhood x chronic pain in adulthood, E) Influence of trouble relaxing on reported chronic pain, F) Association between experiencing muscular symptoms during anxiety episodes and chronic pain

**Appendix C.** **Socio-demographic and health behavior adjusted analysis**

*Expanded methods*

Four socioeconomic variables which had sufficient coverage were available in the dataset: sex (male/female), ever smoked (yes/no), alcohol use status (current, past, never), and age at recruitment (range 40-70 years). These were merged by participant ID to the CRP sample dataset of 2,007 records. Five records with missing ever smoked status were dropped for a total of 2002 records in the modelling dataset. These variables were selected based on a high level of participant responses (~500,000 of the original cases responded on these variables, versus under ~3000 by adding ethnicity filters). Variables such as employment status and ethnicity reduced the case number to a sample too small to warrant analysis, and thus were excluded from this analysis.

A simplified version of the final model was created and compared against a corresponding stratified version with the available socioeconomic variables to identify how socioeconomic factors may affect the results. The simplified model was created by dropping non-significant interactions and variables. The stratified version was fit with a generalized linear mixed-effects model in R, with a Poisson distribution family. For the generalized linear mixed-effects model, sex, ever smoked, and alcohol use were included as fixed effects controls as per standard model building practice (estimated variance on random effects with few levels is imprecise). Age at recruitment was included as a random effect with random intercept (the model intercept was allowed to vary by age at recruitment). In addition, random slopes were included for those dependent variables where an ANOVA indicated the random slope was significant vs the model without.

*Results recap (****Table S4****)*

The generalized linear mixed-effects model results and corresponding simplified final model were built and compared to identify if the available data on socioeconomic factors affected the model results.

- The mixed effects model improves the model fit (AIC 8275 mixed effects vs AIC 8320 GLM)
- The mixed effects control variables ever_smoked, sex, and alcohol drinker are not significant
- All variables which are significant in the simplified final model GLM are still significant in the mixed-effects model
- Coefficient estimates are generally smaller and standard errors larger in the mixed-effects model (e.g. 0.21 vs 0.20 muscle symptoms anxiety, 0.10 vs 0.09 trouble relaxing, etc.), however this is only a very slight difference
  - This suggests conclusions are robust to the available socioeconomic data

**Table S4. Socio-demographic and health behavior adjusted analysis results**

|  | ***Simplified Final Model*** | | | | ***Mixed-Effects Model*** | | | | | | |
| --- | --- | --- | --- | --- | --- | --- | --- | --- | --- | --- | --- |
|  |  | | | | ***Fixed Effects*** | | | | | ***Random Effects (AgeAtRecruit)*** | |
| **Variable** | **β** | **Std. Error** | **z value** | ***P*-value** | **β** | **Std. Error** | **z value** | ***P*-value** | ***Variance*** | | ***Std. deviation*** |
| **(Intercept)** | 0.8587 | 0.0710 | 12.091 | < 0.001 | 0.8930 | 0.0803 | 11.118 | < 0.001 | 0.0174 | | 0.1319 |
| **ProfessionalInfoAnxietyYes** | 0.0249 | 0.0415 | 0.601 | 0.548 | 0.0381 | 0.0561 | 0.680 | 0.496 | 0.0354 | | 0.1882 |
| **TroubleRelaxing** | 0.0961 | 0.0174 | 5.529 | < 0.001 | 0.0888 | 0.0183 | 4.856 | < 0.001 |  | |  |
| **MuscleSymptomsAnxietyYes** | 0.2134 | 0.0373 | 5.719 | < 0.001 | 0.1998 | 0.0497 | 4.024 | < 0.001 | 0.0253 | | 0.1602 |
| **FeltHated** | 0.0530 | 0.0156 | 3.401 | 0.001 | 0.0504 | 0.0159 | 3.160 | 0.002 |  | |  |
| **PhysicallyAbused** | 0.0786 | 0.0200 | 3.930 | < 0.001 | 0.0755 | 0.0269 | 2.805 | 0.005 | 0.0085 | | 0.0924 |
| **SexuallyAbused** | 0.0940 | 0.0266 | 3.527 | < 0.001 | 0.0745 | 0.0274 | 2.720 | 0.007 |  | |  |
| **TakenToDoctorIfNeeded** | -0.0431 | 0.0164 | -2.633 | 0.008 | -0.0405 | 0.0167 | -2.426 | 0.015 |  | |  |
| **C_Reactive_Protein_avg** | -0.0075 | 0.0145 | -0.517 | 0.605 | -0.0034 | 0.0147 | -0.231 | 0.818 |  | |  |
| **PhysicallyAbused X SexuallyAbused** | -0.0854 | 0.0181 | -4.727 | < 0.001 | -0.0743 | 0.0186 | -3.990 | < 0.001 |  | |  |
| **ProfessionalInfoAnxietyYes X C_ReactiveProtein_avg** | 0.0394 | 0.0104 | 3.769 | < 0.001 | 0.0392 | 0.0108 | 3.624 | < 0.001 |  | |  |
| **PhysicallyAbused X C_ReactiveProtein_avg** | -0.0136 | 0.0043 | -3.168 | 0.002 | -0.0135 | 0.0044 | -3.035 | 0.002 |  | |  |
| **SexuallyAbused X C_ReactiveProtein_avg** | -0.0183 | 0.0062 | -2.957 | 0.003 | -0.0156 | 0.0063 | -2.476 | 0.013 |  | |  |
| **TakenToDoctorIfNeeded X C_ReactiveProtein_avg** | 0.0083 | 0.0036 | 2.291 | 0.022 | 0.0066 | 0.0037 | 1.764 | 0.078 |  | |  |
| **PhysicallyAbused X SexuallyAbused X C_ReactiveProtein_avg** | 0.0196 | 0.0044 | 4.502 | < 0.001 | 0.0172 | 0.0045 | 3.808 | < 0.001 |  | |  |
| **ProfessionalInfoAnxietyNo X FeltHated X C_ReactiveProtein_avg** | 0.0029 | 0.0046 | 0.633 | 0.527 | 0.0023 | 0.0047 | 0.490 | 0.624 |  | |  |
| **ProfessionalInfoAnxietyYes X FeltHated X C_ReactiveProtein_avg** | -0.0174 | 0.0046 | -3.750 | < 0.001 | -0.0171 | 0.0049 | -3.491 | < 0.001 |  | |  |
| **EverSmokedYes** |  |  |  |  | -0.0100 | 0.0283 | -0.354 | 0.723 |  | |  |
| **SexMale** |  |  |  |  | -0.0536 | 0.0282 | -1.902 | 0.057 |  | |  |
| **AlcoholDrinkerNever** |  |  |  |  | 0.0631 | 0.0984 | 0.641 | 0.522 |  | |  |
| **AlcoholDrinkerPrevious** |  |  |  |  | 0.1226 | 0.0705 | 1.739 | 0.082 |  | |  |

Simplified final model: Null deviance: 3018 on 2001 degrees of freedom. Residual deviance: 2770 on 1985 degrees of freedom. AIC: 8320.

Mixed-effects model: Deviance: 8213. Residual degrees of freedom: 1971. AIC: 8275.
